# Antegrade or Retrograde Approach for the Management of Tandem Occlusions in Acute Ischemic Stroke: A Systematic Review and Meta-Analysis

**DOI:** 10.3389/fneur.2021.757665

**Published:** 2022-01-12

**Authors:** Xiaoli Min, Jianhua Du, Xuesong Bai, Tao Wei, Adam A. Dmytriw, Aman B. Patel, Xiao Zhang, Xin Xu, Yao Feng, Tao Wang, Xue Wang, Kun Yang, Weiwu Hu, Tingyu Yi, Wenhuo Chen, Liqun Jiao

**Affiliations:** ^1^Department of Cerebrovascular Diseases, The Second Affiliated Hospital, Kunming Medical University, Kunming, China; ^2^Department of Neurosurgery, Xuanwu Hospital, Capital Medical University, Beijing, China; ^3^Peking Union Medical College, Chinese Academy of Medical Sciences, Beijing, China; ^4^China International Neuroscience Institute (China-INI), Beijing, China; ^5^Library, Kunming Medical University, Kunming, China; ^6^Neuroendovascular Program, Massachusetts General Hospital, Harvard Medical School, Boston, MA, United States; ^7^Medical Library, Xuanwu Hospital, Capital Medical University, Beijing, China; ^8^Department of Evidence-Based Medicine, Xuanwu Hospital, Capital Medical University, Beijing, China; ^9^Department of Neurology, The First Traditional Chinese Medicine Hospital of Chengde, Chengde, China; ^10^Department of Neurology, Zhangzhou Affiliated Hospital, Fujian Medical University, Fuzhou, China; ^11^Department of Interventional Neuroradiology, Xuanwu Hospital, Capital Medical University, Beijing, China

**Keywords:** antegrade approach, retrograde approach, tandem occlusions, acute ischemic stroke, meta-analysis

## Abstract

**Background:** Acute ischemic stroke (AIS) caused by tandem intracranial and extracranial occlusions is not rare. However, optimal strategy between antegrade (extracranial first) or retrograde (intracranial first) approaches still remains elusive. This systematic review and meta-analysis aim to compare the two approaches to provide updated clinical evidence of strategy selection.

**Methods:** PubMed, Ovid, Web of Science, and the Cochrane Library were searched for literature comparing antegrade and retrograde approaches for patients with AIS with concomitant tandem occlusions. Outcomes including successful reperfusion [Throbolysis in Cerebral Infarction (TICI) 2b−3] and 90-day favorable outcome [modified Rankin Scale (mRS) 0–2], any intracerebral hemorrhage, symptomatic intracerebral hemorrhage, procedural complications, and mortality were evaluated. The risk of bias was assessed using the Newcastle–Ottawa Scale and illustrated in the Funnel plot. Heterogeneity was assessed by *I*^2^ statistic. Subgroup and sensitivity analyses were also performed.

**Results:** A total of 11 studies accounting 1,517 patients were included. 831 (55%) patients were treated with an antegrade approach and 686 (45%) patients were treated with the retrograde approach. A higher successful reperfusion rate was achieved in retrograde group than that of antegrade group [83.8 vs. 78.0%; odds ratio (OR): 0.63, 95% CI: 0.40–0.99, *p* = 0.04]. 90-day favorable outcome (mRS 0–2 at 90 days) also showed significantly higher in retrograde group compared with antegrade group (47.3 vs. 40.2%; OR: 0.72, 95% CI: 0.58–0.89, *p* = 0.002). The incidence of any intracranial hemorrhage (ICH), symptomatic intracranial hemorrhage, 90-day mortality, and other complications did not differ between two groups.

**Conclusion:** In AIS with tandem occlusions, the retrograde approach might achieve a higher successful reperfusion rate and better functional outcome with a comparable safety profile when compared with an antegrade approach. Further prospective controlled studies with more meticulous design and a higher level of evidence are needed to confirm these results.

**Systematic Review Registration:** “PROSPERO” database (CRD 42020199093), https://www.crd.york.ac.uk/PROSPERO/.

## Introduction

Acute ischemic stroke (AIS) is a major contributor to global morbidity and mortality (1). AIS caused by tandem occlusions, including a proximal extracranial occlusion in conjunction with an intracranial occlusion is not rare and was reported to consist of 10–20% of all the patients with stroke (1, 2). But, the treatment for this unique subgroup of patients is challenging. Achieving successful reperfusion is still the primary goal of treatment and will increase the likelihood of favorable functional outcomes. Previous studies have shown that the rate of reperfusion *via* intravenous tissue plasminogen activator (IV tPA) alone may be unsatisfactory (3, 4). Recently, the superiority of mechanical thrombectomy (MT) over IV tPA was established by clinical evidence from a series of randomized controlled trials (RCTs) (1, 5, 6). Thus, MT has been the first-line choice of treatment.

For AIS with tandem occlusions, MT plus emergent carotid artery stenting could achieve a considerably high chance of reperfusion and functional independence (7, 8) and thus, has been considered as an acceptable treatment modality (9, 10). Nevertheless, evidence for optimal order of treatment, including antegrade (extracranial first) and retrograde (intracranial first) approach, is still elusive and controversial. Although comparable outcomes between the two approaches were found in the previous meta-analysis by Wilson et al. (11) the majority of recruited studies were single armed lacking direct comparison data. Also, many studies with larger sample size and directly comparing the two approaches were published thereafter (12–15). Some studies still showed comparable results between the two approaches (13, 14). In stark contrast, a better outcome of the retrograde approach was shown in some other studies (12, 15). Additionally, the retrograde approach was adopted as the prior choice in some centers with respect to its effectiveness and safety (14, 16). Therefore, it is necessary to summarize the current literature comparing the two approaches, thus providing updated clinical evidence of strategy selection and decision-making for this special group of patients.

## Methods

### Search Strategy and Eligibility Criteria

This study is conducted according to the Preferred Reporting Items for Systematic Reviews and Meta-Analyses (PRISMA) guidelines (see online Supplementary Files 1, 2. PRISMA Checklist) (17). A literature search was performed to identify studies assessing patients of AIS caused by tandem lesions with treatment strategy of antegrade (extracranial first) or retrograde (intracranial first) approaches. We searched the following databases including PubMed, Ovid, Web of Science, and the Cochrane Library for eligible studies. The preliminary search terms, including stroke, large-vessel occlusion, acute ischemic stroke, tandem occlusion, internal carotid artery (ICA) occlusion, and MT, were utilized to capture all the studies eligible for inclusion. The search strategy was detailed in the supplementary material (see online Supplementary File 3. Search strategy).

Two reviewers (YF and XZ) screened and selected the eligible studies independently. Any disagreements were resolved with the help of another more senior reviewer (XB). Studies were regarded as eligible if they met the following criteria: reporting both the antegrade and retrograde approaches for patients with AIS, with ≥ 70% stenosis or occlusion of the extracranial ICA and concomitant occlusion of the intracranial ICA and/or proximal middle cerebral artery (MCA); outcomes data were sufficient and reported separately, so as to compare the two approaches: RCTs and observational studies (cohort studies and case–control studies). Studies were ruled out if duplicates and review articles, no comparison between antegrade and retrograde approaches, insufficient information, patients were not eligible, only abstract published, and case reports. The antegrade approach is defined as proximal-to-distal revascularization, with angioplasty and/or stenting of the extracranial lesion first, followed by intracranial thrombectomy, while the retrograde approach is defined as distal-to-proximal revascularization, with the treatment of the intracranial occlusion with MT first, followed by treatment of the extracranial ICA occlusion.

### Baseline and Outcome Data Extraction

After the selection of studies, a standardized form was used for data extraction by one author (XZ) and rechecked by another author (YF). The main characteristics of included studies were extracted including authors and publication year, recruitment period, country and centers, study design, total sample size, number of each group of the patients, etiology, therapy strategy, and favorable strategy. The following baseline data from each study were collected: age, gender, the National Institutes of Health Stroke Scale (NIHSS) score at admission, the Alberta Stroke Program Early CT Score (ASPECTS) at admission, the proportion of patients treated with IV thrombolysis, site of occlusion, thrombectomy technique, and procedural duration (defined as the mean/media time from a puncture to final reperfusion).

Available data of outcome variables were also extracted including successful reperfusion (defined as TICI 2b−3 after endovascular treatment); favorable outcome/functional independence (defined as mRS 0–2 at 90 days); symptomatic intracerebral hemorrhage (sICH) [defined as ICH on imaging and a minimum increase of four points on the NIHSS within 24 h postintervention in accordance with the second European-Australasian Acute Stroke Study (ECASS II) classification (18)]; any ICH (defined as symptomatic ICH or asymptomatic ICH); procedural complications (defined as any procedure- and device-related complication such as artery dissection, artery perforation, clot migration, embolization, stent deployment failure, or device malfunction); and mortality at 90 days.

### Assessment of Heterogeneity and Publication Bias

Heterogeneity was measured with the *I*^2^ statistic and the value of *I*^2^ > 50% was regarded as moderate-to-high heterogeneity depending on the pooled results (19). Subgroup analysis was conducted to explore the reasonable origins of heterogeneity on the condition that the results with high heterogeneity and a sufficient number of included trials. We tried to perform subgroup analysis based on publication year or ethnicity. Sensitivity analysis was also conducted to evaluate the effect of exclusion for the study with a high overall risk bias.

To qualitatively evaluate the risk of publication bias, two independent reviewers (XM and JD) assessed each article through the Newcastle–Ottawa scale (NOS), which was designed for observational studies quality assessment (see online Supplementary File 4. NOS scale) (20). Each domain of included studies was given a score on the risk of bias (see online Supplementary File 5. Study quality assessment). Then, quantitatively evaluated publication bias by the Begg's test and the Egger's test and funnel plot was illustrated in case of the included studies exceed 10 (21).

### Statistical Analysis

Odds ratio (OR) with 95% CIs were used to depict the dichotomous data of most baseline and outcome variables and mean differences (MDs) with 95% CIs for continuous variables such as mean age, the NIHSS or the ASPECTS score, and mean time of puncture to reperfusion. Statistical significance is defined at *p* < 0.05. Only in the case of there being sufficient effect size or sample size (at least three studies), outcomes data were pooled in meta-analysis. The software Revman 5.3 (The Nordic Cochrane Centre, Copenhagen, Denmark) and RStudio software (R-Tools Technology, Inc., Canada): the Nordic Cochrane Center; https://rstudio.com/products/rstudio. (version 4.0.4) (RStudio using “meta” and “metafore” packages) were used for statistical analyses.

## Results

### Selection and Characteristics of Studies

**Figure 1:**
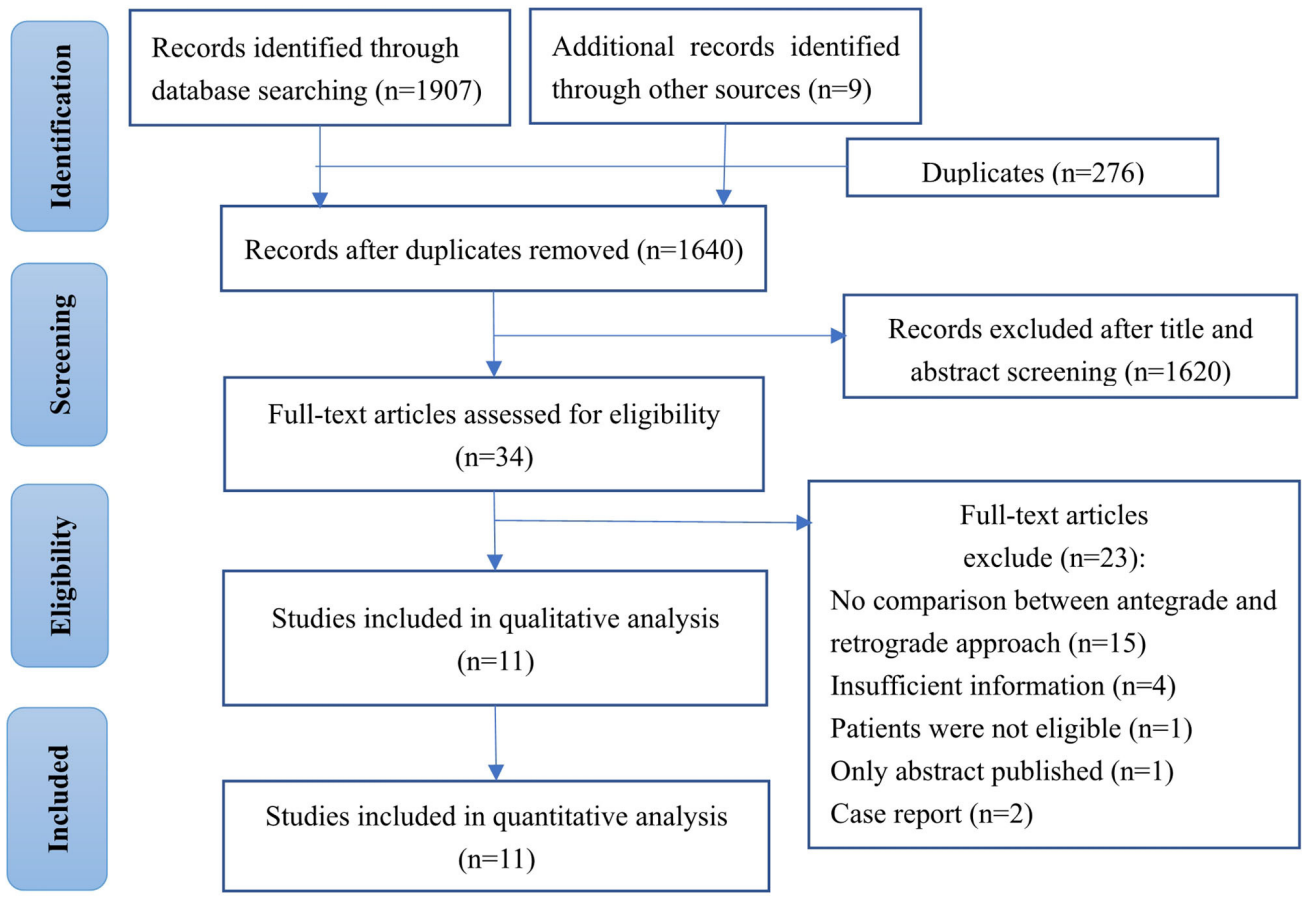
Flow diagram of literature for systematic review and meta-analysis.

A total of 1,907 articles were initially identified through main electronic database and references of related studies, of which 34 full-text articles were retrieved for assessment after duplicates were removed and title/abstract screened. Eventually, 11 studies that met our inclusion criteria were eligible for final analysis (12–15, 22–28). All the included studies had more than one outcome indicator available that was presented separately for comparison analysis between the different treatment strategies. The selection process of studies and reasons for exclusion were detailed in a flow diagram (Figure 1).

The main characteristics of included studies and baseline data of patients were given in Tables 1, 2 and Supplementary File 6. All the studies were published between 2015 and 2021. Among them, five studies were retrospective single-center studies, one study was retrospective multicenter studies, and the remaining five studies were large multicenter cohort with retrospective review from the prospective collected database. Meanwhile, three studies were conducted in Asia country and the other eight studies were carried out by a research collaboration mainly in Europe or USA. A total of 1,517 patients (sample size from 17 to 493) with AIS and concomitant anterior circulation tandem lesion were enrolled including 831 (55%) patients who adopted antegrade approach (extracranial treatment first or proximal-to-distal revascularization) and 686 (45%) patients who adopted retrograde approach (intracranial treatment first or distal-to-proximal revascularization) as endovascular therapy strategies. Interestingly, the retrograde approach was more favorable in 7 out of these 11 included studies. Mean age of patients ranged from 58.7 to 75.2 years and the majority gender was male (71.9%). The median NIHSS score at admission and the ASPECT score were ranged from 12 to 18 and 7 to 9, respectively. The intracranial occlusion site is mostly located at the terminal furcation of ICA and M1 or M2 segment of MCA. The proportion of patients bridging IV tPA ranged from 27.3 to 100%. Mean time of puncture to final reperfusion regarded as procedural duration spanned from 33 to 130 min. In addition, the rate of TICI 2b−3 successful reperfusion and mRS 0–2 favorable outcome were 80.6% (50–92.1%) and 43.5% (30–71.4%), respectively. Any procedure- and device-related complications including arterial dissection, artery perforation, clot migration, embolization in new territory, and stent deployment failure or device malfunction were reported in six studies and the incidence of any of these complications was 17.7% (6.5–24.9%). The incidence of sICH complication was 8.1% (5–16.1%), while mortality risk was 16.7% (6.9–32.3%).

**Table 1:** Characteristics of included studies.

**References**	**Country; Centers**	**Study** **design**	**Etiology** **(n)**	**Sample size**	**Patients number,** **n, AGA/RGA**	**Advocated strategy**
Lockau et al. (22)	Germany; Single center	RC	AS (24); AD (13)	37	12/25	RGA
Puri et al. (23)	USA; Multicenter	RC	AS (20); AD (8)	28	24/4	AGA
Moptsaris et al. (24)	Germany; Single center	RC	AS (52); AD (9)	63	17/46	NA
Eker et al. (14)	France; Multicenter	RPC	AS (121)	121	46/75	RGA
Maus et al. (15)	Germany; Multicenter	RPC	AS (144); AD (21)	171	101/70	RGA
Yang et al. (12)	China; Multicenter	RC	AS (40); AD (11); CE (8); other (1)	60	31/29	RGA
Luu et al. (27)	Vietnam; Single center	RC	NA	17	10/7	RGA
Neuberger et al. (13)	Germany; Single center	RPC	NA	162	85/77	NA
Park et al. (26)	Korea; Single center	RC	AS (76)	76	56/20	NA
Feil et al. (28)	Germany; Multicenter	RPC	NA	493	267/226	RGA
Haussen et al. (25)	USA; Multicenter	RPC	AS (289)	289	182/107	RGA

*RC, retrospective cohort; RPC, retrospective analysis of prospective collected data; AS, arteriosclerosis; AD, arterial dissection; CE, Cardioembolism; AGA, Antegrade approach; RGA, Retrograde approach; NA, not available*.

**Table 2:** Main baseline data of included patients.

**Study**	**Group (n)**	**Age** **(years)**	**Male,** **n(%)**	**Admission** **NIHSS**	**Admission** **ASPECTS**	**IV tPA,** **n(%)**	**Procedural** **duration (min)**
Lockau et al. (22)	AGA (12) RGA (25)	64 (36–89) 62 (40–84)	7 (58.3) 20 (80.0)	17 (3–30) 17 (5–27)	NA	6 (50.0) 14 (56.0)	130.2 ± 45.1 58.6 ± 26.1
Puri et al. (23)	AGA (24) RGA (4)	58.7 (30–83)	19 (67.8)	18 (2–28)	NA	7 (27.3)	NA
Moptsaris et al. (24)	AGA (17) RGA (46)	67 (33–84)	49 (78)	14 (1–29)	NA	33(52)	110 (15–208) 125 (45–212)
Eker et al. (14)	AGA (46) RGA (75)	70 (63–79) 70 (63–76)	31 (67.4) 59 (78.7)	18 (13–21) 16 (10–19)	NA	NA	95 (53–141) 82 (61–109)
Maus et al. (15)	AGA (101) RGA (70)	64 ± 12 67 ± 12	66 (65) 52 (74)	15 ± 6 16 ± 6	NA	65 (64) 41 (59)	103 ± 46 123 ± 56
Yang et al. (12)	AGA (31) RGA (29)	64 (60–70) 60 (41–68)	24 (77.4) 27 (93.1)	NA	NA	8 (25.8) 13 (44.8)	125 (86–167) 116 (91–179)
Luu et al. (27)	AGA (10) RGA (7)	70.5 ± 10.7 69.9 ± 6.1	10 (100) 6 (85.7)	15.2 (7–20) 18.6 (14–24)	7.4 ± 0.8 7.9 ± 1.3	3 (30) 7 (100)	82.2 ± 37.9 55.4 ± 15.8
Neuberger et al. (13)	AGA (85) RGA (77)	73.3 ± 11.5	124 (76.5)	16 (11–20)	NA	NA	NA
Park et al. (26)	AGA (56) RGA (20)	70.5 ± 10.9 75.2 ± 11.1	46 (82) 17 (85)	12 (8–15) 13 (7–15)	8 (7–10) 9 (8–10)	NA	36 (25–55) 33 (27–40)
Feil et al. (28)	AGA (267) RGA (227)	69.3 ± 11.9 67.5 ± 11.2	184 (68.9) 163 (71.8)	14 (9–18) 14 (10–18)	8 (7–10) 8 (7–10)	145 (54.3) 141 (62.1)	72 (50–101.5) 53.5 (36–78)
Haussen et al. (25)	AGA (182) RGA (107)	67.2 ± 10.5 65.9 ± 10.5	119 (65.4) 68 (64.2)	15.7 ± 5.9 15.8 ± 6.2	8 (7–10) 7 (6–8)	112 (61.5) 63 (58.9)	70 (50–102) 56 (39–90)

*Data was expressed as mean ± SD, median (IQR), or n(%)*.

*SD, standard deviation; IQR, interquartile range; AGA, Antegrade approach; RGA, Retrograde approach; NIHSS, National Institute of Health Stroke Scale; ASPECTS, Alberta Stroke Program Early CT Score; IV tPA, intravenous tissue plasminogen activator; NA, not available*.

### Main Outcomes Comparison Between Antegrade and Retrograde Approaches

**Figure 2:**
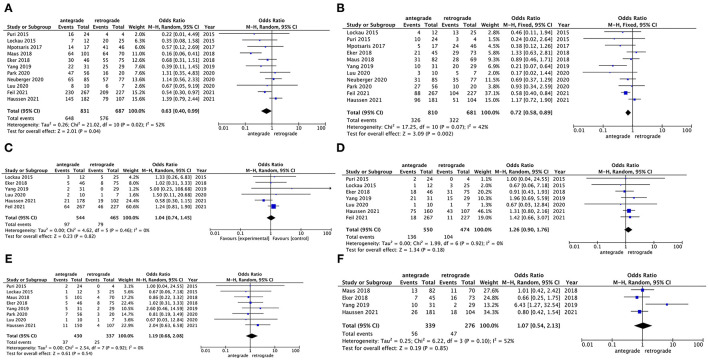
Forest plot showing the comparison for main outcomes between the antegrade and retrograde groups. **(A)** successful reperfusion (TICI 2b−3); **(B)** favorable outcome (90-day mRS 0–2); **(C)** any procedural complication; **(D)** any intracranial hemorrhage; **(E)** symptomatic intracranial hemorrhage; and **(F)** 90-day mortality.

Meta-analysis for main outcomes of antegrade and retrograde groups is shown in Table 3 and Forest plot is given correspondingly in Figure 2. All the 11 studies provided data on the TICI 2b−3 representing successful reperfusion and the pooled results indicated that a slightly higher rate of successful reperfusion in the retrograde group than that in antegrade group (83.8 vs. 78.0%; OR: 0.63, 95% CI: 0.40–0.99, *p* = 0.04) (Figure 2A). Notably, with *I*^2^ value more than 50% (*p* = 0.02, *I*^2^ = 52%), high heterogeneity existed among studies. Sensitivity analysis was further performed and indicated that heterogeneity might origin from two articles published by author Maus et al. and Haussen et al., however, both were high quality studies, which launched by international multicenter collaborations with a relatively large sample size. At the same time, procedural duration from original literature is given in Table 2, but not enrolled ultimately in meta-analysis due to the inappropriate data type of continuous variables. Even so, seven included studies reported procedural duration. The mean time from a puncture to final reperfusion also showed a bit shorter time advantage tendency of retrograde approach at a rough estimate (retrograde: range from 33 to 123 min vs. antegrade: range from 36 to 130 min). There was no publication bias according to the Begg's test (*p* = 0.10) and the Egger's test (*p* = 0.41), which is visualized in the Funnel plot (see online Supplementary File 7. Publication bias assessment).

**Table 3:** Pooled meta-analysis outcomes of antegrade and retrograde approaches.

**Outcomes**	**No. of studies**	**AGA (%)**	**RGA (%)**	**OR (95% CI)**	***I*** **^2^ (%)**	***P*** **-value**
Successful reperfusion (TICI 2b−3)	11	78.0	83.8	0.63 (0.40–0.99)	52	0.040
Favorable outcome (90-day mRS 0–2)	11	40.2	47.3	0.72 (0.58–0.89)	42	0.002
Any procedure related complication	6	17.8	17.0	1.04 (0.74–1.45)	0	0.820
Any ICH	7	24.7	21.9	1.26 (0.90–1.76)	0	0.180
sICH	8	8.6	7.4	1.19 (0.68–2.08)	0	0.540
90-day Mortality	4	16.5	17.0	1.07 (0.54–2.13)	52	0.850

*AGA, Antegrade approach; RGA, Retrograde approach; TICI, Thrombolysis in Cerebral Infarction; mRS, modified Rankin Scale; sICH, symptomatic intracranial hemorrhage; ICH, intracrinial hemorrhage; OR, odds ratio; CI, confidence interval; I^2^, the variation attributable to heterogeneity*.

Favorable outcome also reported by all the 11 studies and the pooled results indicated that the retrograde group achieved a significantly better 90-day functional independence than the antegrade group (47.3 vs. 40.2%; OR: 0.72, 95% CI: 0.58–0.89, *p* = 0.002) (Figure 2B). Sensitivity analysis indicated the heterogeneity (*p* = 0.07, *I*^2^ = 42%) also coming from the aforementioned two articles by Maus et al. (15) and Haussen et al. (25). No publication bias (Begg's test: *p* = 0.31; Egger's test: *p* = 0.17) was observed (see online Supplementary File 7. Publication bias assessment).

Procedure- and device-related complications were reported in six studies. Four studies provided the data on artery dissection or perforation, two studies gave the data on clot migration or embolization, and three studies demonstrated the data on stent deployment failure or device malfunction. The incidence of any procedural complication was 17.0% in the retrograde group, while the incidence of any procedural complication was 17.8% in the antegrade group. The pooled results showed that there was no significant difference between the two treatment strategies (OR: 1.04, 95% CI: 0.74–1.45, *p* = 0.82) (Figure 2C) and no heterogeneity (*p* = 0.46, *I*^2^ = 0%) was observed. The incidence of any ICH complication was provided by seven studies. The pooled results indicated a tendency that the retrograde group might have a lower incidence of hemorrhage complication than the antegrade group, but this tendency was not significant (21.9 vs. 24.7%; OR: 1.26, 95% CI: 0.90–1.76, *p* = 0.18) (Figure 2D). When considering sICH, eight studies provided the relative data and pooled results showed a similar tendency (7.4 vs. 8.6%; OR: 1.19, 95% CI: 0.68–2.08, *p* = 0.54) (Figure 2E). No inspected significant heterogeneity (*p* = 0.18, *I*^2^ = 0%; *p* = 0.92, *I*^2^ = 0%) was also there. Publication bias was not performed due to limited data with respect to these complications.

90-day mortality was reported in four studies and the incidence of mortality in the retrograde group and the antegrade group was 17.0 and 16.5%, respectively. There was no remarkable difference in mortality risk between the two treatment strategies observed in pooled results (OR: 1.07, 95% CI: 0.54–2.13, *p* = 0.85) (Figure 2F). However, a high heterogeneity (*p* = 0.10, *I*^2^ = 52%) was detected and the heterogeneity might origin from the article published by Yang et al. (12). Publication bias was not performed due to limited data.

### Subgroup Analyses of Outcomes Based on Heterogeneity Origin

**Figure 3:**
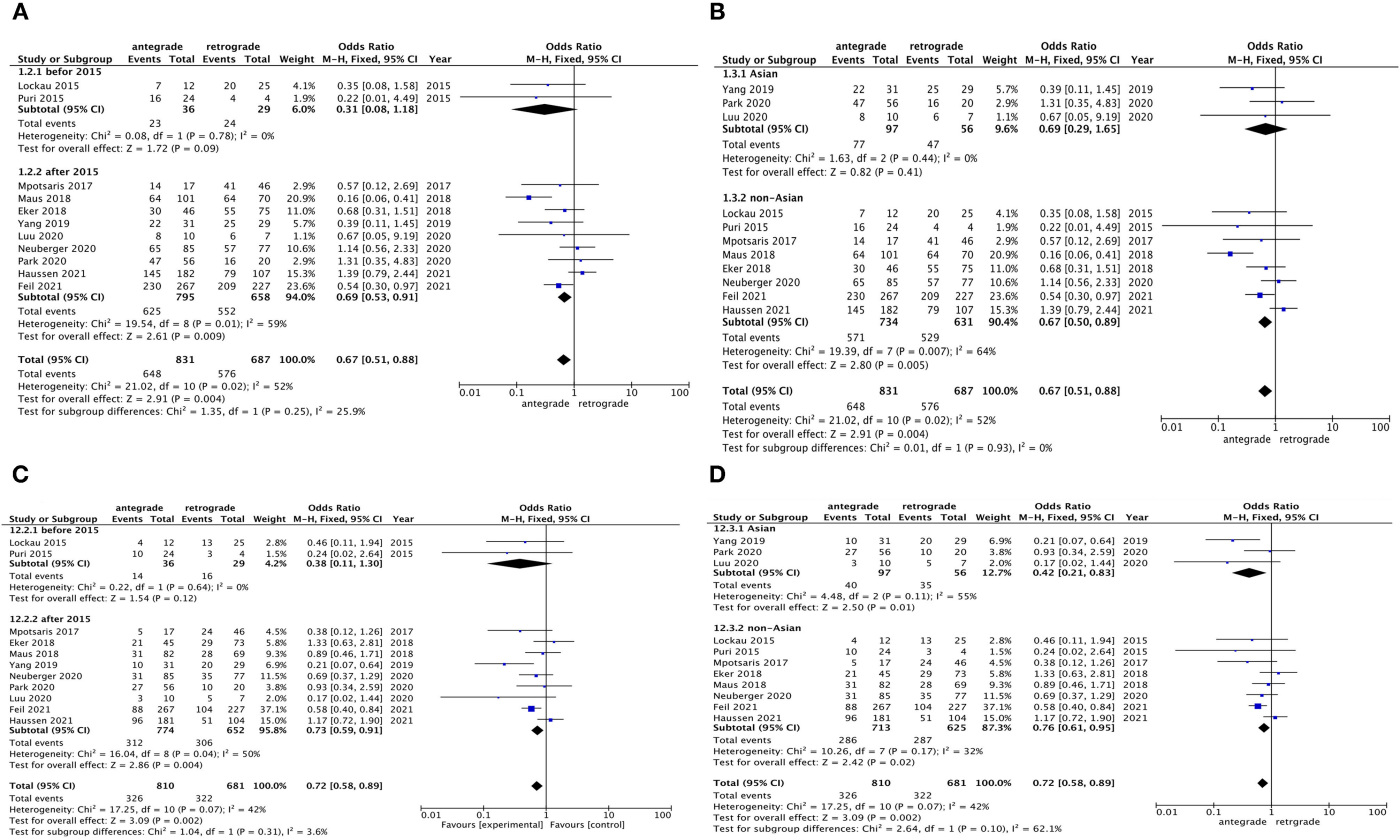
Subgroup analysis for heterogeneous outcomes of antegrade and retrograde groups based on article published year and the region of data source of the patients. **(A,B)** successful reperfusion (TICI 2b−3) and **(C,D)** favorable outcomes (90-day mRS 0–2).

According to sensitivity analysis clue, we found that the publication year (before or after 2015) and ethnicity (Asian or non-Asian) might be the potential source of high heterogeneity. Subgroup analysis for outcomes of successful reperfusion between antegrade and retrograde groups was conducted by publication year or ethnicity, separately. The results showed that studies published after 2015 (*p* = 0.01, *I*^2^ = 59%) and within the non-Asian subgroup (*p* = 0.007, *I*^2^ = 64%) still existed high heterogeneity (Figures 3A,B). Meanwhile, for favorable outcome, subgroup analysis result of studies published after 2015 (*p* = 0.04, *I*^2^ = 50%) and the results within both the Asian (*p* = 0.11, *I*^2^ = 55%) and non-Asian subgroups (*p* = 0.17, *I*^2^ = 32%) also showed moderate-to-high heterogeneity (Figures 3C,D). Subgroup analysis of outcomes with respect to 90-day mortality and hemorrhage or procedural complications was not performed due to sensitivity analysis negative results or limited data.

## Discussion

This systematic review and meta-analysis compared antegrade with retrograde approaches in the treatment of AIS due to tandem lesions accounting for over 1,000 patients. Pooled results showed that the retrograde approach treating intracranial occlusion first then extracranial lesion might achieve a higher successful reperfusion rate and, thus, a better 90-day functional outcome than antegrade approach (successful reperfusion: 83.8 vs. 78.0%; favorable outcome: 47.3 vs. 40.2%). The incidence of any ICH and sICH was a statistically non-significant trend toward numerically lower in retrograde approach than that of antegrade approach (any ICH: 21.9 vs. 24.7%; sICH: 7.4 vs. 8.6%). Furthermore, there was no statistical difference in either procedure-related complication rate (17.0 vs. 17.8%) or 90-day mortality risk (17.0 vs. 16.5%) between the two strategies.

Tandem occlusion, including a proximal extracranial high-grade stenosis or occlusion in conjunction with a downstream intracranial occlusion, is not rare for patients with AIS. Currently, Endovascular Intervention (EVT) has been proved to be an effective technique for treating tandem occlusion by increasing evidence (29). However, tailored evidence for the optimal treatment order between antegrade and retrograde approaches remained inconsistent and contradictory among studies, as both the approaches have innate advantages and disadvantages. The advantage of the antegrade approach is to provide blood flow to the non-occluded arteries and prevents possible recurrent occlusion of the intracranial vessels due to slow or stagnant flow. Also, it may allow bigger guide catheters for safer access to distal lesions. During distal clot removal with stent retrievers, interventionalist can easier advance the balloon guide catheter for proximal “flow arrest.” In some extreme cases where the retrograde approach is not feasible due to high-grade stenosis, the antegrade approach seems to be the only choice. The major disadvantage of the retrograde approach is that intracranial thrombectomy prior to extracranial occlusion treatment may expose patients to the potential higher risk of distal embolism, especially when lacking the use of distal protection devices. However, there are opposite results showing that even a higher rate of distal embolism occurred in the antegrade approach (15). This might be explained by that foreword blood flow restoration after proximal occlusion relief increased pressure on intracranial thrombus, which resulted in clot fragments into distal terminal vessel branches. Additionally, the antegrade approach treating cervical stenosis or occlusion first might delay the intracranial reperfusion due to time-consuming. In the contrast, the advantage of the retrograde approach is a quicker restoration of cerebral blood flow to the intracranial ischemia area and hypothetically can reduce cerebral ischemia.

Our finding suggested that the retrograde approach might be superior to the antegrade approach with respect to more and earlier blood restoration and better functional outcome achievable. This was in line with several recent large sample size multicenter studies demonstrating faster distal occlusion reperfusion consistently in better clinical outcomes with the retrograde approach (25, 28). Primarily, revascularization of the proximal extracranial lesion with Percutaneous Endovascular Angioplasty (PTA) and/or stenting before distal intracranial occlusion treatment seemed to be time-consuming thus postponed intracranial reperfusion. This disadvantage could be avoided in the retrograde approach. Once the microwire and catheter could navigate through the proximal artery lesion segment without difficulty, intracranial thrombectomy was conducted immediately that would shorten the ischemic time of attacked brain territory. Another possible reason was owing to the rapid development of new thrombectomy devices represented by stent retriever and large bore suction bolt catheter. Compared to older devices, the new generation devices could reduce the technical difficulty and guarantee to achieve a higher reperfusion rate at the shorter procedural time (30). Nevertheless, the higher rate of successful reperfusion with the retrograde approach seems hard to explain and needs to be further tested. There have been studies showing time-dependent clot composition changes and there is possible preferential degradation of the erythrocyte-rich part of the clot in the early stage. Delayed thrombectomy is, thus, more likely to face the clots with a higher proportion of fibrin and platelets, which are associated with later passes of thrombectomy and, thus, increase the difficulty of reperfusion (31). Retrograde approach may help to decrease the risk of clot distal embolism by flushing out the small thrombi with primarily restored intracranial foreword blood flow during extracranial occlusion treatment.

A higher favorable outcome rate was another important observation in this study, with putative beneficial from a higher rate of successful reperfusion. Successful reperfusion after acute large vessel occlusive stroke was a robust predictor associated with good clinical outcomes (32). For AIS due to tandem occlusions, distal vascular revascularization may be more important than the proximal lesion processing. Thus, the treatment order of the retrograde approach allowed the intracranial offending vessel to gain rapid reperfusion contributing to the anoxia and ischemic brain tissue rescue in the affected territory. Early reperfusion shortens the ischemic duration and could bring more beneficial for poor or not yet established leptomeningeal collateral circulation in the acute phase of stroke. This might be the partially pathophysiologic explanation for favorable functional independence corresponding to successful reperfusion. Moreover, extracranial stent angioplasty prior to intracranial thrombectomy was hypothesized to increase additional procedure- and device-related complications due to scratching between thrombectomy device and carotid stent struts, especially in case of advancing resistance (33, 34). Retrograde approach, through adopting the intracranial first strategy, could shun these potential risks associated with deteriorating clinical outcomes.

Concerning the safety profile between antegrade and retrograde approaches, our meta-analysis showed no statistic differences in terms of procedural or hemorrhagic complications as well as 90-day mortality, except for the trend toward a lower rate of any ICH and sICH in patients treated with the retrograde approach. In the latest large sample size registry study, a comparison of intracranial- and extracranial-first approaches in more than 600 patients demonstrated no statistical differences in periprocedural complications, but a slightly lower incidence of periprocedural complications with the intracranial-first approach (28). Our results were in line with this study and other previous reports (12, 25). However, our results were inconsistent with the respective analysis demonstrating retrograde approach had higher periprocedural complications than antegrade approach (23), but the results were based on small sample size and heterogeneous populations. A previous meta-analysis observed no statistic differences of outcomes parameters and safety profiles between intracranial- and extracranial-first group, except for a slightly lower procedure-related complication (11). The optimal technical approach still needs clarification in prospective studies with meticulous design, although related RCT seems to be impractical.

This study has some limitations. First, we could not rule out the inherent risk of selection bias due to respective observational design, despite approximately a half of studies coming from prospective collected database. Heterogeneity among studies might be inevitable, thus subgroup analysis was performed on putative heterogeneity derivations. Also, several outcomes were not enrolled in meta-analysis due to the inappropriate type such as continuous variables or data limited. There were rare literature specifying cervical stenosis *vs*. complete occlusion with respect to its impact on revascularization strategies and pathophysiology, so that the inadequate data prevented a meta-analysis from being performed, as cases with extremely severe stenosis may influence the decision of revascularization strategies and probably lead to a bias of the final outcomes. A similar condition existed with respect to distal embolization. However, the strengths of this meta-analysis are recruiting studies with a direct comparison of the two approaches. There are several studies with multicenter design and large sample size. Compared to the previous meta-analysis, this study will provide updated clinical evidence of strategy selection and decision-making for AIS with tandem occlusions and will definitely be helpful in guiding future clinical trials.

## Conclusion

In conclusion, for AIS due to tandem occlusions, the retrograde approach might achieve a higher successful reperfusion rate and better favorable functional outcome with a comparable safety profile when compared with the antegrade approach. Further prospective controlled studies with more meticulous design and a higher level of evidence are warranted.

## Data Availability Statement

The original contributions presented in the study are included in the article/Supplementary Material, further inquiries can be directed to the corresponding authors.

## Author Contributions

XB and LJ contributed to the initial idea for this study. XM, TWe, XZ, WH, and TWa developed and revised the search strategy. XM, XB, XZ, XW, and JD completed the study design. WC and LJ contributed to consults about clinical issues. YF and XZ extracted the data. KY analyzed the extraction data. XM, XZ, and XB contributed to the original draft. XB, XX, TY, WC, and LJ contributed to the revision of the draft. XM and JD qualitatively evaluated the risk of publication bias. All authors approved the final manuscript prior to submission.

## Funding

This study was supported by the Natural Science Foundation of China (No. 81960219) and the Clinical Research Project of the Second Affiliated Hospital of Kunming Medical University (No. 2020ynlc009).

## Conflict of Interest

The authors declare that the research was conducted in the absence of any commercial or financial relationships that could be construed as a potential conflict of interest.

## Publisher's Note

All claims expressed in this article are solely those of the authors and do not necessarily represent those of their affiliated organizations, or those of the publisher, the editors and the reviewers. Any product that may be evaluated in this article, or claim that may be made by its manufacturer, is not guaranteed or endorsed by the publisher.
