# Supplementary material for: Antegrade or Retrograde Approach for the Management of Tandem Occlusions in Acute Ischemic Stroke: A Systematic Review and Meta-Analysis
Source: Front Neurol. 2022 Jan 12;12:757665. doi: 10.3389/fneur.2021.757665 (PMC8790816; doi:10.3389/fneur.2021.757665)
Supplement: Supplementary file 3 [file Table_3.DOCX]

- **Ovid MEDLINE(R) and Epub Ahead of Print, In-Process & Other Non-Indexed Citations, Daily and Versions(R) from January 01, 2015 to May 01, 2021**

| **No.** | **Searches** |
| --- | --- |
| 1 | cerebrovascular disorders/ or basal ganglia cerebrovascular disease/ or exp brain ischemia/ or exp brain infarction/ or exp carotid artery diseases/ or carotid artery thrombosis/ or intracranial arterial diseases/ or cerebral arterial diseases/ or infarction, anterior cerebral artery/ or infarction, middle cerebral artery/ or infarction, posterior cerebral artery/ or exp “intracranial embolism and thrombosis”/ or exp stroke/ |
| 2 | (isch?emi$ adj5 (stroke$ or apoplex$ or cerebral vasc$ or cerebrovasc$ or brain vasc$ or cva or attack$)).tw. |
| 3 | ((brain or cerebr$ or cerebell$ or vertebrobasil$ or hemispher$ or intracran$ or intracerebral or infratentorial or supratentorial or middle cerebr$ or MCA or anterior circulation or basilar artery or vertebral artery) adj5 (isch?emi$ or infarct$ or thrombo$ or emboli$ or occlus$ or hypoxi$)).tw. |
| 4 | 1 or 2 or 3 |
| 5 | carotid artery, internal/ |
| 6 | carotid artery thrombosis/ or carotid stenosis/ or arterial occlusive diseases/ or exp arteriosclerosis/ or constriction, pathologic/ |
| 7 | 5 and 6 |
| 8 | ((internal carotid or ICA or tandem) adj5 (stenos?s or occlus$ or occlud$ or thrombo$ or narrow$ or plaque$ or constrict$ or emboli$ or block$ or arteriosclero$ or atherosclero$ or atheroma$ or isch?emi$ or infarct$ or insufficien$ or obstruct$)).tw. |
| 9 | 7 or 8 |
| 10 | endovascular procedures/ or catheterization/ or angioplasty/ or exp angioplasty, balloon/ |
| 11 | vascular surgical procedures/ or exp thrombectomy/ or exp embolectomy/ |
| 12 | exp stents/ |
| 13 | (angioplast$ or stent$ or pta or revasculari?ation or recanali?ation or catheter$ or dilatation or thromboaspirat$ or thrombo-aspirat$ or thrombecto$ or embolecto$).tw. |
| 14 | ((clot or thrombus or thrombi or embol$) adj5 (aspirat$ or remov$ or retriev$ or fragmentation or retract$ or extract$ or obliterat$ or dispers$)).tw. |
| 15 | ((mechanical or pharmacomechanical or endovascular or neurovascular) adj5 (thrombolys$ or reperfusion or fragmentation or aspirat$)).tw. |
| 16 | thrombolytic therapy/ or fibrinolytic agents/ or tissue plasminogen activator/ or exp plasminogen activators/ or fibrinolysis/ |
| 17 | (thromboly$ or fibrinoly$ or recanali?ation).tw. |
| 18 | ((clot or thrombus or thrombi or embol$) adj5 (lyse or lysis or dissolve$ or dissolution)).tw. |
| 19 | (tPA or t-PA or rtPA or rt-PA or plasminogen or plasmin or alteplase or actilyse).tw. |
| 20 | (anistreplase or streptodornase or streptokinase or urokinase or pro?urokinase or rpro?uk or lumbrokinase or duteplase or lanoteplase or pamiteplase or reteplase or saruplase or staphylokinase or streptase or tenecteplase or desmoteplase or retevase).tw. |
| 21 | 16 or 17 or 18 or 19 or 20 |
| 22 | infusions, intra-arterial/ |
| 23 | (intra arterial or intra-arterial or intraarterial or IA).tw. |
| 24 | 22 or 23 |
| 25 | 21 and 24 |
| 26 | 10 or 11 or 12 or 13 or 14 or 15 or 25 |
| 27 | 4 and 9 and 26 |
| 28 | randomized controlled trials as topic/ |
| 29 | randomized controlled trial/ |
| 30 | random allocation/ |
| 31 | double blind method/ |
| 32 | single blind method/ |
| 33 | clinical trial/ |
| 34 | clinical trial, phase i.pt |
| 35 | clinical trial, phase ii.pt |
| 36 | clinical trial, phase iii.pt |
| 37 | clinical trial, phase iv.pt |
| 38 | controlled clinical trial.pt |
| 39 | randomized controlled trial.pt |
| 40 | multicenter study.pt |
| 41 | clinical trial.pt |
| 42 | exp clinical trials as topic/ |
| 43 | or/28-42 |
| 44 | (clinical adj trial$).tw |
| 45 | ((singl$ or doubl$ or treb$ or tripl$) adj (blind$3 or mask$3)).tw |
| 46 | placebos/ |
| 47 | placebo$.tw |
| 48 | randomly allocated.tw |
| 49 | (allocated adj2 random$).tw |
| 50 | or/44-49 |
| 51 | 43 or 50 |
| 52 | case report.tw |
| 53 | letter/ |
| 54 | historical article/ |
| 55 | or/52-54 |
| 56 | 51 not 55 |
| 57 | exp case control studies/ |
| 58 | exp cohort studies/ |
| 59 | case control.tw. |
| 60 | (cohort adj (study or studies)).tw. |
| 61 | cohort analy$.tw. |
| 62 | (observational adj (study or studies)).tw. |
| 63 | longitudinal.tw. |
| 64 | retrospective.tw. |
| 65 | cross sectional.tw. |
| 66 | cross-sectional studies/ |
| 67 | or/57-66 |
| 68 | 56 or 67 |
| 69 | exp animals/ not humans.sh. |
| 70 | 68 not 69 |
| 71 | 27 and 70 |
| 72 | limit 71 to english language |
