# Supplementary material for: Antegrade or Retrograde Approach for the Management of Tandem Occlusions in Acute Ischemic Stroke: A Systematic Review and Meta-Analysis
Source: Front Neurol. 2022 Jan 12;12:757665. doi: 10.3389/fneur.2021.757665 (PMC8790816; doi:10.3389/fneur.2021.757665)
Supplement: Supplementary file 5 [file Table_5.DOCX]

**Supplementary file 5. Table. The New Castle-Ottawa scoring for cohort studies.**

| **Study** | **Subject Selection Max 4** | **Study Comparability Max2** | **Assessment of Outcomes Max3** | **Total Score** | **Risk of bias** |
| --- | --- | --- | --- | --- | --- |
| Lockau et al, 2015^23^ | 4 | 0 | 3 | 7 | Low |
| Puri et al, 2015^24^ | 3 | 0 | 3 | 6 | Low |
| Moptsaris et al, 2017^3^ | 4 | 0 | 3 | 7 | Low |
| Eker et al, 2018^9^ | 4 | 0 | 2 | 6 | Low |
| Maus et al, 2018^25^ | 4 | 0 | 2 | 6 | Low |
| Yang et al, 2019^25^ | 4 | 0 | 3 | 7 | Low |
| Luu et al, 2020^25^ | 4 | 0 | 2 | 6 | Low |
| Neuberger et al, 2020^25^ | 4 | 0 | 3 | 7 | Low |
| Park et al, 2020^25^ | 4 | 0 | 3 | 7 | Low |
| Feil et al, 2021^25^ | 4 | 0 | 3 | 7 | Low |
| Haussen et al, 2021^25^ | 4 | 0 | 3 | 7 | Low |
