# Supplementary material for: Antegrade or Retrograde Approach for the Management of Tandem Occlusions in Acute Ischemic Stroke: A Systematic Review and Meta-Analysis
Source: Front Neurol. 2022 Jan 12;12:757665. doi: 10.3389/fneur.2021.757665 (PMC8790816; doi:10.3389/fneur.2021.757665)
Supplement: Supplementary file 6 [file Table_6.DOCX]

**Supplementary file 6. Table. Additional baseline data of included patients.**

| Study | Group (n) | Lesion site (n) | Thrombectomy technique (n) | Successful reperfusion,  n（%） | Favorable outcome,  n（%） | Any ICH,  n（%） | sICH, n（%） | Procedural complications,  n（%） | 90-day Mortality,  n（%） |
| --- | --- | --- | --- | --- | --- | --- | --- | --- | --- |
| Lockau et al, 2015^22^ | AGA(12)  RGA(25) | ICA-T（5）,M1/M2（7）;  ICA-T（11）,M1/M2（14） | NA | 7(58.3)  20(80.0) | 4(33.3)  13(52.0) | NA | 1(8.3)  3(12.0) | 3(24.9)  5(16.0) | NA |
| Puri et al, 2015^23^ | AGA(24)  RGA(4) | NA | NA | 14(50) | 16(56.5) | NA | 2(7.1) | NA | 4(14) |
| Moptsaris et al, 2017^24^ | AGA(17)  RGA(46) | ICA-T（19）  M1/M2（44） | NA | 14(82)  41(89) | 29(46) | NA | 3(5) | NA | 8(13) |
| Eker et al, 2018^14^ | AGA(46)  RGA(75) | NA | NA | 30(65.2)  55(73.3) | 21(46.7)  29(39.7) | 18(39.2)  31(41.4) | 5(10.9)  8(10.7) | 5(10.9)  8(10.7) | 7(15.6)  16(21.9) |
| Maus et al, 2018^15^ | AGA(101)  RGA(70) | ICA-T（33）,M1/M2（68）;  ICA-T（17）,M1/M2（53） | NA | 64(63)  64(91) | 31(38)  28(41) | NA | 5(5)  4(6) | NA | 13(16)  11(16) |
| Yang et al, 2019^12^ | AGA(31)  RGA(29) | NA | AC（1）,SR（20）;  AC（0）,SR（21） | 22(71.0)  25(86.2) | 10(32.3)  20(69.0) | 21(67.7)  15(51.7) | 5(16.1)  2(6.9) | 2(6.5)  0(0) | 10(32.3)  2(6.9) |
| Luu et al, 2020^27^ | AGA(10)  RGA(7) | ICA-T（3）,M1/M2（7）;  ICA-T（3）,M1/M2（4） | AC（4）,SR（4）,  Both (2);  AC（0）,SR（6）,  Both (1) | 8(80)  6(85.7) | 3(30)  5(71.4) | NA | 1(10)  1(14.3) | 2(20)  1(14.3) | NA |
| Neuberger et al, 2020^13^ | AGA(85)  RGA(77) | NA | NA | 65(76.4)  57(74.2) | 31(36.5)  35(45.3) | 58(35.8) | 15(9.3) | NA | 20(23.5)  9(11.3) |
| Park et al, 2020^26^ | AGA(56)  RGA(20) | ICA-T（17）,M1/M2（39）;  ICA-T（6）,M1/M2（14） | NA | 47(84)  16(80) | 27(48)  10(50) | NA | 7(13)  3(15) | NA | NA |
| Feil et al, 2021^28^ | AGA(267)  RGA(227) | ICA-T（94）,M1/M2（172）;  ICA-T（68）,M1/M2（157） | AC（14）,SR（107）,  Both (133);  AC（17）,SR（74）,  Both (132) | 230(86.1)  209(92.1) | 88(32.9)  104(45.8) | 68(25.5)  49(21.6) | NA | 64(24.0)  46(20.3) | NA |
| Haussen et al, 2021^25^ | AGA(182)  RGA(107) | ICA-T（61）,M1/M2（30）;  ICA-T（121）M1/M2（77） | NA | 145(79.7)  79(73.8) | 96(53.0)  51(49.0) | 75(46.9)  43(40.2) | 11(6.9)  4(3.7) | 21(11.8)  19(18.6) | 26(14.4)  18(17.3) |

Data was expressed as mean±SD, median(IQR), or n(%)

Abbreviations: SD, standard deviation; IQR, interquartile range; AGA, Antegrade approach; RGA, Retrograde approach; ICA-T, terminal internal carotid artery; M1/M2, M1 or M2 segment of middle cerebral artery; AC, aspiration catheter; SR,stent retriever;NA,not available;ICH, intracranial cerebral hemorrhage; sICH, symptomatic intracranial cerebral hemorrhage.Procedural duration is defined as the mean/media time from groin puncture to final recanalization;successful reperfusion is defined as TICI 2b-3 after endovascular treatment; favorable outcome is defined as mRS 0-2 at 90 days.
