# Supplementary material for: Antegrade or Retrograde Approach for the Management of Tandem Occlusions in Acute Ischemic Stroke: A Systematic Review and Meta-Analysis
Source: Front Neurol. 2022 Jan 12;12:757665. doi: 10.3389/fneur.2021.757665 (PMC8790816; doi:10.3389/fneur.2021.757665)
Supplement: Supplementary file 7 [file Table_7.DOCX]

A


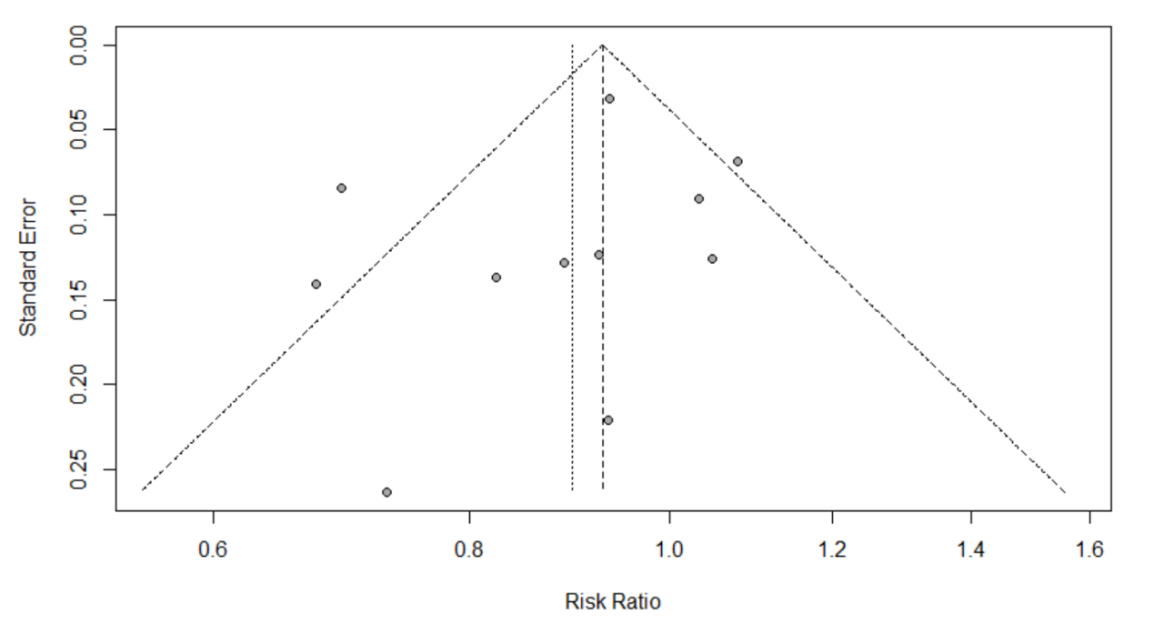


B


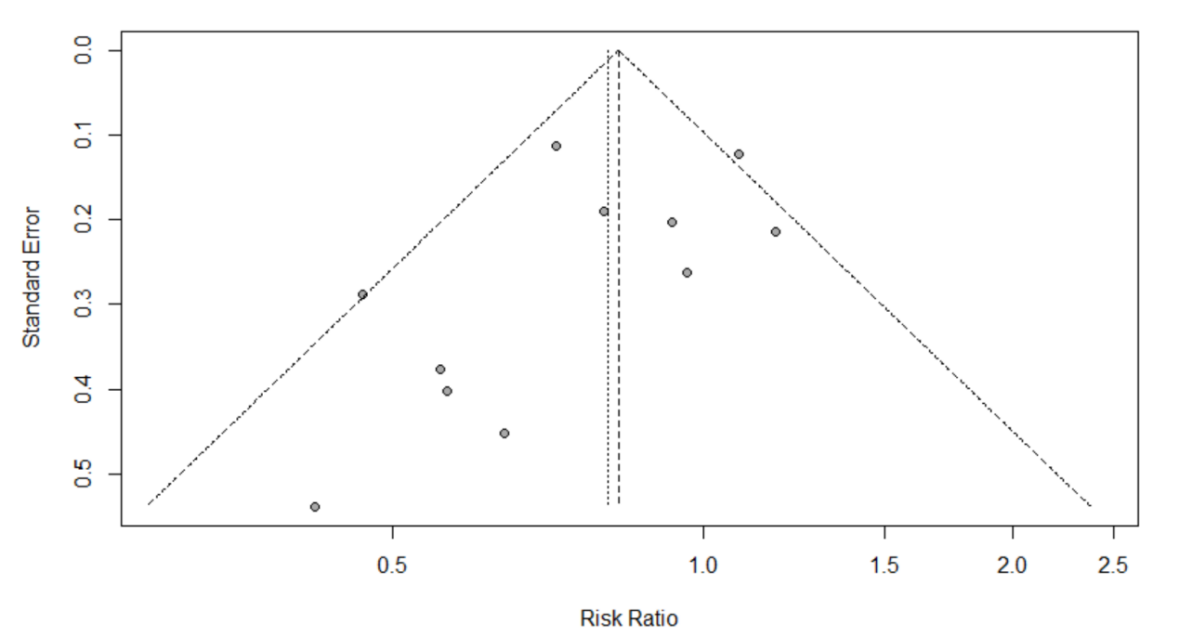


**Supplementary file 7** funnel plots for visualizing publication bias on studies. (A) successful reperfusion(TICI 2b-3); (B)favorable outcome(90-day mRS 0-2).
